# Effect of bridge height on airflow and aeolian sand flux near surface along the Qinghai-Tibet Railway, China

**DOI:** 10.1038/s41598-024-66647-0

**Published:** 2024-07-10

**Authors:** Chengjie Xue, Kecun Zhang, Zhishan An, Jianhua Xiao, Hongxue Zhang, Jiapeng Pan

**Affiliations:** 1 Key Laboratory of Ecological Safety and Sustainable Development in Arid Lands / Dunhuang Gobi Desert Ecology and Environment Research Station, Northwest Institute of Eco-Environment and Resources, Chinese Academy of Sciences, Lanzhou, 730000 China; 2 University of Chinese Academy of Sciences, Beijing, 100049 China

**Keywords:** Railway bridge, Height, Flow field structure, Sand accumulation, Numerical simulation, Natural hazards, Mathematics and computing

## Abstract

In this work, we studied the near-surface flow field structure of railway bridges with different heights through field investigation and wind tunnel simulation experiments. Meanwhile, we simulated the distribution of sand accumulation around a bridge via CFD software based on the sand accumulation around the Basuoqu bridge in the Cuona Lake section of the Qinghai–Tibet Railway. Results show that the sand around this railway bridge is mainly from the lake sediment on the west side of the railway and the weathered detritus on the east side. The height of the railway bridge in the sandy area affects the distribution of the near-surface flow field and the variation in speed on both sides of the bridge. The wind speed trough on both sides of the 6 m high bridge is higher, and the horizontal distance between the wind speed trough and the bridge section is 1.5 times that of the 3 m high bridge. Wind speed attenuates in a certain range on the windward and leeward sides of the bridge, forming an aeolian area; under the beam body, it is affected by the narrow tube effect, forming a wind erosion area. The height of the bridge determines its sand transport capacity. Under certain wind conditions, the overhead area at the bottom of the 3 m high bridge and its two sides do not have the sand transport capacity, so sand accumulates easily. Nevertheless, the sand accumulation phenomenon gradually disappears with the increase in bridge clearance height. The objectives of this study are to reveal the formation mechanism of sand damage for railway bridges, provide theoretical support for the scientific design of railway bridges in sandy areas, and formulate reasonable railway sand prevention measures to ensure the safety of railway running, which have certain theoretical significance and practical value.

## Introduction

The Qinghai–Tibet Railway is the highest and longest plateau railway in the world, with a total length of 1956 km, passing through various geomorphic units, including the Gobi, desert, snowy mountains, grasslands, swamps, wetlands, and frozen areas^1^ The natural environment along the Gobi and quicksand areas through which the Qinghai–Tibet Railway passes is harsh, and the wind is strong^2^. In addition, the disturbance of the local natural environment during the construction process breaks the balance of the original ecological system, and the railway is vulnerable to sand damage, such as subgrade erosion and sand burial. Wind and sand disasters have become the main obstacle to the safe operation of the Qinghai–Tibet Railway. Therefore, disaster control is of great importance. According to the preliminary investigation, the total length of the sand-damaged sections along the Qinghai–Tibet Railway is 200 km, and the serious sections are 43 km, which are mainly concentrated in Xidatan, Wudaoliang, Beiluhe Basin, Qingshuihe, and Cuona Lake. Aiming at the problem of sand damage on the Qinghai–Tibet Railway^3,4^, researchers have realized certain scientific achievements in the characteristics of the sand flow field, sand damage formation mechanism, and sand control measures through field monitoring, wind tunnel experiments, numerical simulation, and changes in soil physical and chemical properties, thus ensuring the safe operation of the Qinghai–Tibet Railway.

Previous studies have paid more attention to the cost, feasibility and economic benefits of railway construction, but less attention has been paid to the relationship between the height of railway bridges and the wind-blown sand process. The design height of a railway bridge in sandy areas is important, which not only affects the cost of railway construction and operation but also restricts the local wind–sand transport process on both sides of the railway and the effectiveness of the wind–sand protection system. Before the construction of a railway bridge, the transport of near-surface wind-blown sand is in a relatively balanced state. After the construction, the bridge interferes with the local wind field, breaking the original stable dynamic balance of wind-blown sand transport and changing the spatial distribution pattern of wind-blown sand flow passing through the bridge. If the design height of a bridge is inadequate, it can result in sand accumulation around the bridge and even block the flow channels at its base, posing a direct threat to railway safety^5^. There has been limited research on the impact of bridge height on flow field structure and sand accumulation^6,7^. Accordingly, researching the effects of bridge height, analyzing the disturbance caused by bridge height on the wind–sand flow field, and clarifying the formation mechanism of sand damage are necessary.

Based on field observation and wind tunnel simulation experiment, this study analyzes the influence of railway bridges with different heights on the surrounding flow field structure and uses the Euler two-fluid model to simulate the distribution of sand accumulation around bridges, reveal the formation mechanism of railway bridge sand damage under different environments, and provide a theoretical basis for the formulation of railway sand control measures. The research results could help further reveal the formation mechanism and development trend of sand damage to railway bridges and road engineering in similar areas and provide a scientific basis for formulating reasonable prevention and control measures.

## Study area

The Cuona Lake section of the Qinghai–Tibet Railway extends for approximately 2.0 km, with the railway bridge placed about 3.0 m above the surface of the lake. Active dunes around this section are widely developed, mainly barchan dunes under the action of a single dominant wind direction. The survey found that the migration rate of active dunes in this section is more than 2 m/a. The harmfulness of dunes is mainly manifested as wind erosion, wind deposition, and their combined action^8^.

**Figure 1:**
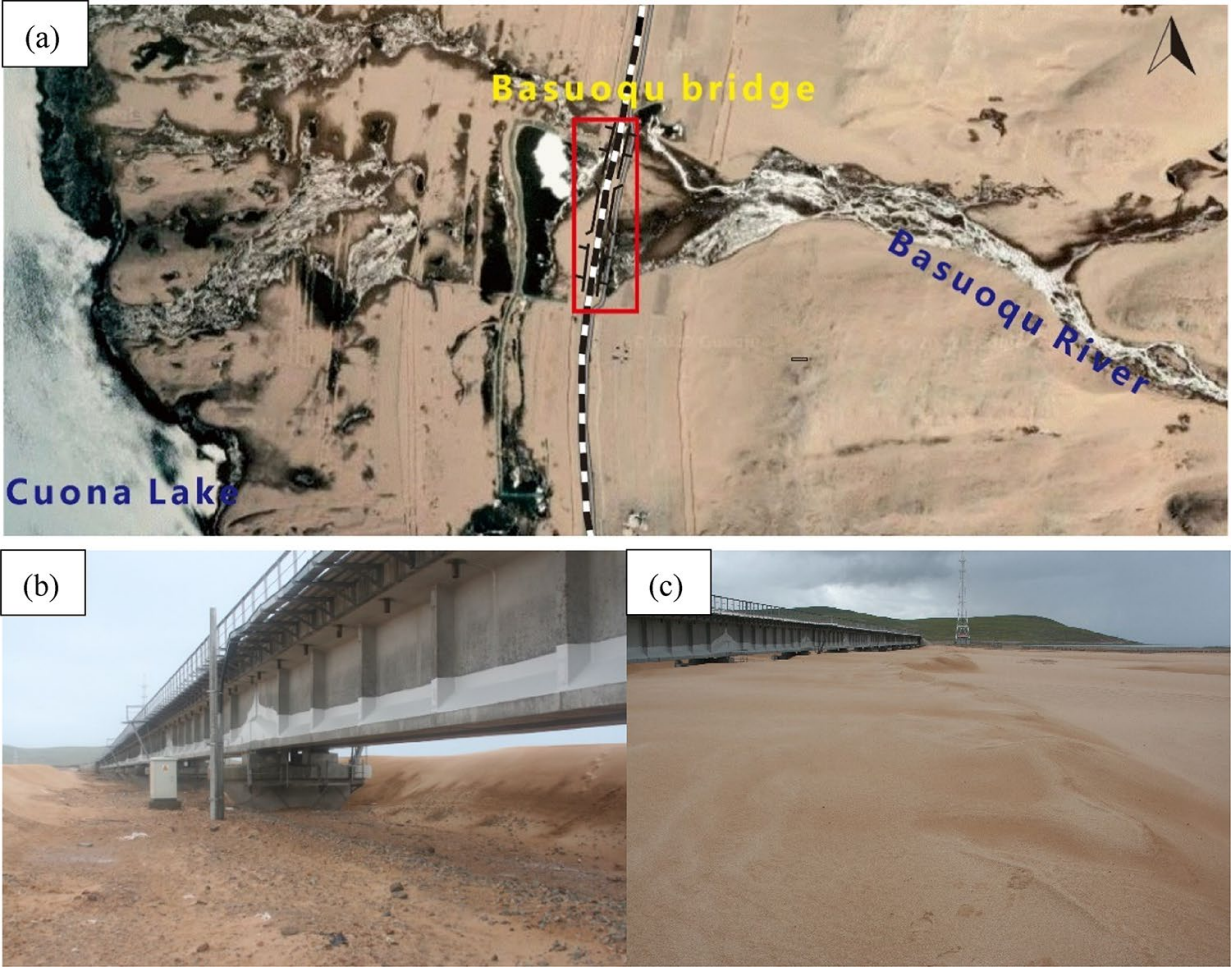
Sand accumulation on the railway bridge of the Cuona Lake section of the Qinghai-Tibet Railway (**a**: Image from Amap, Amap version:13.17.0.2012, https://ditu.amap.com).

Basuoqu bridge is located on the east side of Cuona Lake (Fig. 1a). The east side of the bridge is weathered debris of hills, and the west side is lakeside desertification grassland. In the summer, substantial amounts of weathered debris from the hills east of the railway are transported into the lake by water runoff. During winter, the lake's surface freezes, leading to a considerable shrinkage, exposing abundant in sand sources around the lake's vast area. During the wind season, sand source is transported and deposited in the piedmont plain. The aeolian sand in this section has the characteristics of circulation. When aeolian sand flow encounters obstacles, the cross section of aeolian sand flow changes, the air flow energy decreases, the sand carrying capacity decreases, and aeolian sand is formed. Sand damage of Basuoqu bridge is due to the bridge breaking the original balance of the surrounding wind-sand flow field, forming a typical wind-accumulated sand damage.

Basuoqu bridge is a low-clearance bridge (Fig. 1b), and the accumulation of sand on both sides of the bridge has a tendency to develop towards the clearance area at the bottom of the bridge, which will further block the bottom of the beam, cause river interruption, wind sand onto the rail, and seriously affect traffic safety. In some areas, the accumulated sand is close to the bottom of the beam (Fig. 1c). In recent years, a large number of sand prevention projects have been implemented, but the problem of sand damage still exists.

## Research methods

### Dynamic environment of wind-sand activity

**Figure 2:**
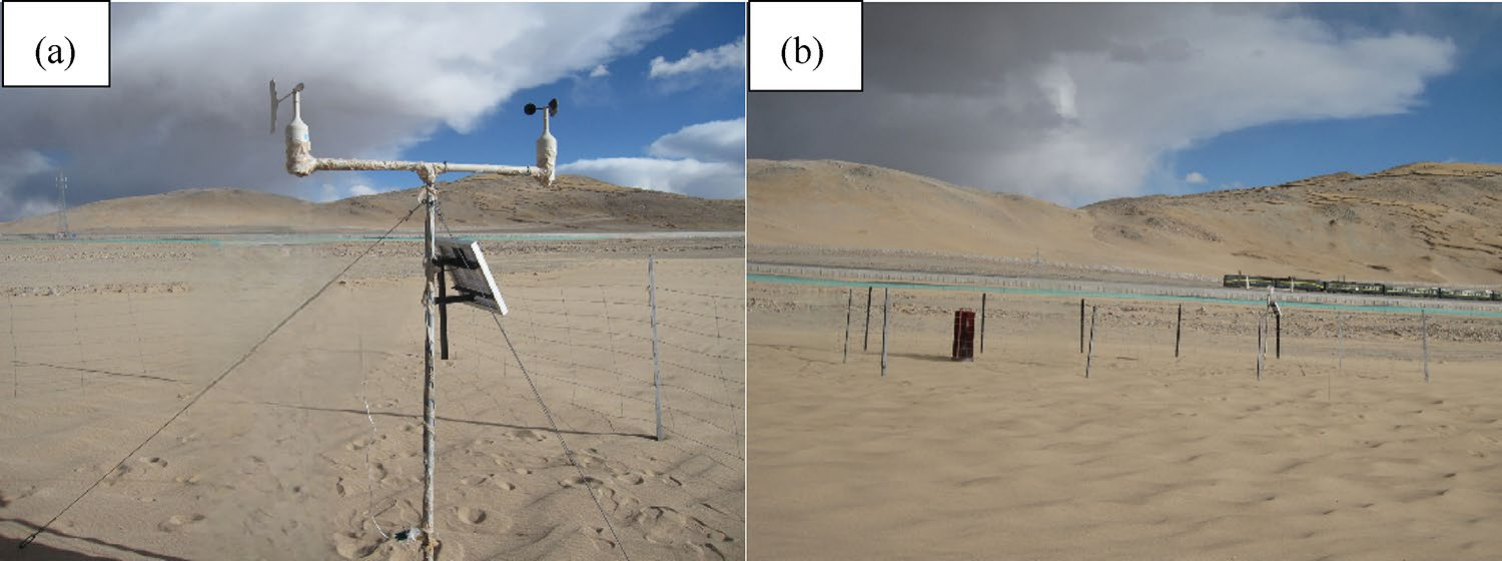
Observation experiment sites and instruments.

The research data are from the wind speed and direction observation data obtained by the HOBO automatic meteorological instrument and the sand accumulation data obtained by the sand collecting instrument on the west side of the bridge. The HOBO automatic meteorological instrument records the average data every 10 min, and the observation height is 2 m from the ground (Fig. 2a).

Sand transport potential is one of the important indicators to measure the intensity of sand activity in the region, and it represents the potential sand transport capacity, that is, the sand transport capacity of a certain direction of the wind speed statistics in a certain time, which is expressed in vector unit (VU). At present, the most widely used method to calculate the sediment transport potential is the Leto equation adopted by Fryberger^9,10^.

1 $$DP = V^{2} \left( {V - Vt} \right) \, t,$$

where DP is the sediment transport potential (vector unit "VU"); V is the wind speed (m/s); Vt is the sand-driving wind speed (m/s), and the sand-driving wind speed is uniformly 5 m/s^11–13^. t is the action time of sand-driving wind, which is expressed as frequency (%) in the statistical table.

According to the method of superposition of the 16-direction vector of sediment transport potential, the synthesized direction is called the synthesized sediment transport direction (RDD), which represents the net direction of sediment transport. The resultant sediment transport direction is called the resultant sediment transport potential (RDP), which represents the net sediment transport potential under various wind directions.

### Wind tunnel simulation experiment

**Figure 3:**
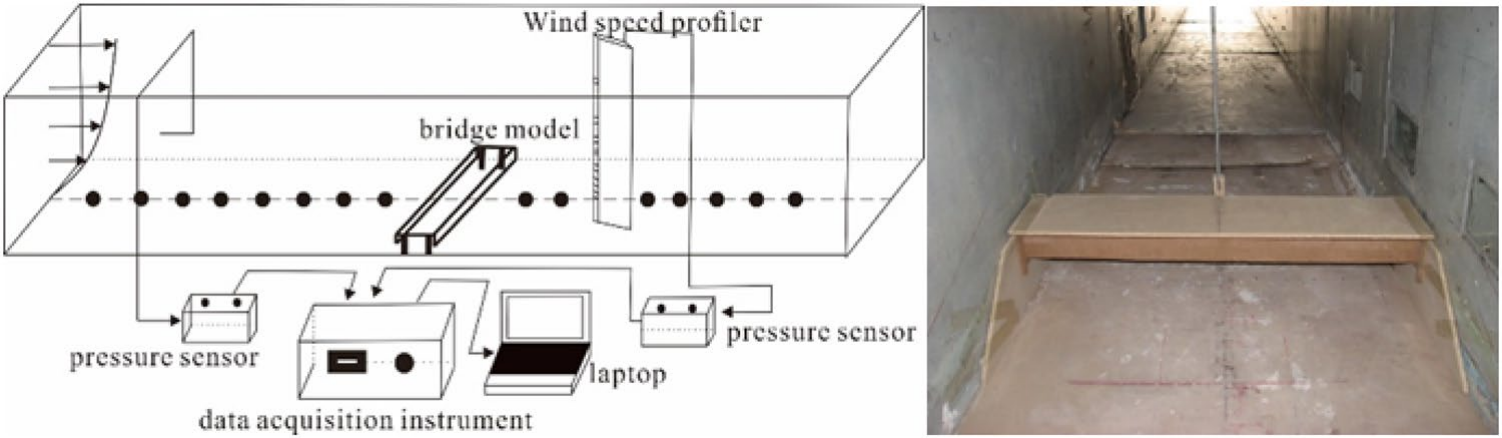
Schematic diagram of experimental layout.

This experiment was conducted in the field wind tunnel of the Key Laboratory of Desert and Desertification, Chinese Academy of Sciences. The wind tunnel is 40 m long and consists of four parts: power section, rectification section, experimental section, and diffusion section. The cross-section of the wind tunnel is 1.2 m × 1.2 m^14,15^. The boundary layer thickness of the wind tunnel is 40 cm. The specific experimental model layout is shown in Fig. 3.

The railway bridge in the Cuona Lake section of the Qinghai–Tibet Railway was selected for the simulation experiment. The top surface width of the bridge was about 4 m, the beam body thickness was about 1.8 m, and the headroom at the bottom of the beam was about 3 m. According to *China’s Code for Railway Line Design(TB 10098-2017)* and the field investigation, bridge heights of 3 m and 6 m were selected for the experiment. In the meanwhile, bridge’s model was scaled at 1:20 combined with boundary layer thickness, the blocking rate of the wind tunnel in this experiment is 9%.

**Figure 4:**
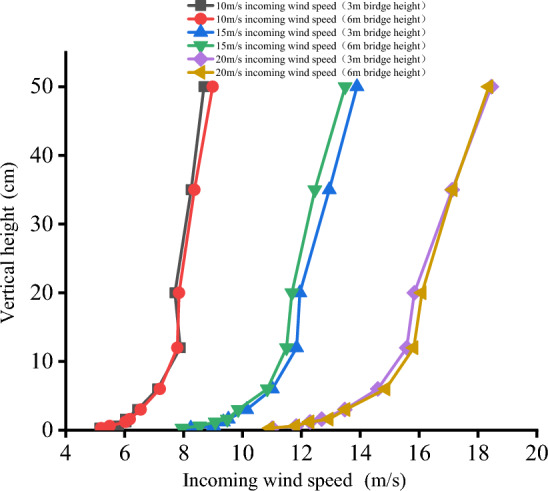
Incoming wind speed profile of wind tunnel.

As the wind direction in the study area is mainly west wind, a single orthogonal wind direction is selected for wind tunnel experiment. Most of the sand-driving wind speed in this region is distributed in 10 – 20 m/s, three kinds of incoming wind speeds were set, which were 10, 15 and 20 m/s in sequence. The incoming wind velocity profile meets the experimental requirements (Fig. 4).

The flow field data were obtained by the wind speed profile set up along the ground in sequence at the central section of the tunnel, and the heights of the wind speed measuring points were 0.3, 0.6, 1.0, 1.5, 3.0, 6.0, 12.0, 20.0, 35.0, and 50.0 cm above the surface. In the horizontal direction, the simulated bridge median line was taken as the benchmark. The wind speed profiler was arranged along the central line of the bottom surface of the tunnel 8H, 7H, 6H, 5H, 4H, 2H, 1H, and 0.5H away from the front and back sides of the bridge, as well as in the front, middle, and back of the bridge, where “H” is the height of the bridge deck, represented by “-H” on the windward side and “ + H” on the leeward side^16–20^.

### Numerical simulation

#### Geometric model and meshing

**Figure 5:**
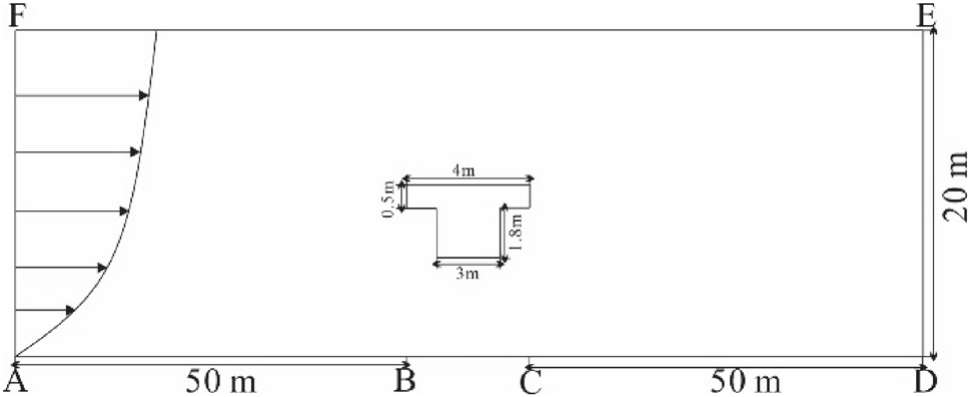
Calculation domain.

Given that the aeolian sand flow is mainly subjected to horizontal and vertical forces during its movement, a two-dimensional model was adopted for simulation analysis. To match the flow field and sand accumulation around the actual bridge, geometric modeling of the railway bridge was carried out at a 1:1 ratio (Fig. 5). The calculation domain size was 20 × 104 m. The meshing type was Quad/Tri, which is a mixture of quadrilateral and triangular meshing. The meshing method was Pave, that is, the area was divided into unstructured meshing, and the number of meshing was 106,000.

#### Basic equation

The maximum flow velocity studied in this paper is 20 m/s < 50 m/s, which can be regarded as an incompressible fluid, and the influence of gravity is taken into account.

(1) Mass conservation equation.

For incompressible fluid motion, where density is constant, the expression is as follows:

2 $$\frac{\partial }{\partial t}({\alpha }_{q}{\rho }_{q})+\nabla \cdot ({\alpha }_{q}{\rho }_{q}{\overrightarrow{v}}_{q})=0,$$

where ${\alpha }_{q}$ represents the volume fraction of the first phase. Wind-blown sand flow is a gas–solid two-phase flow, so the sum of the volume fraction of the gas phase and the sand phase is 1, α_g_ + α_s_ = 1; ${\rho }_{q}$ is the density of the phase $q$; ${\overrightarrow{v}}_{q}$ is the vector velocity of the phase $q$.

(2) Momentum conservation equation.

Gas phase:

3 $$\frac{\partial }{\partial t}({\alpha }_{g}{\rho }_{g}\overrightarrow{{v}_{g}})+\nabla \cdot ({\alpha }_{g}{\rho }_{g}{\overrightarrow{v}}_{g}{\overrightarrow{v}}_{g})=-{\alpha }_{g}{\nabla }_{p}+\nabla \cdot \overline{\overline{{\tau }_{g}}}+{\alpha }_{g}{\rho }_{g}\overrightarrow{g}+{f}_{sg}.$$

Sand grain phase:

4 $$\frac{\partial }{\partial t}({\alpha }_{s}{\rho }_{s}\overrightarrow{{v}_{s}})+\nabla \cdot \left({\alpha }_{s}{\rho }_{s}{\overrightarrow{v}}_{s}{\overrightarrow{v}}_{s}\right)=-{\alpha }_{s}{\nabla }_{p}-{\nabla }_{{p}_{s}}+\nabla \cdot \overline{\overline{{\tau }_{s}}}+{\alpha }_{s}{\rho }_{s}\overrightarrow{g}+{f}_{sg},$$

where $\overrightarrow{g}$ is the acceleration of gravity; $p$ is the same pressure shared by the two phases; ${p}_{s}$ is the pressure of the sand grain phase; $\overline{\overline{{\tau }_{g}}}$ and $\overline{\overline{{\tau }_{s}}}$ are the stress–strain tensors of the gas phase and the sand phase respectively; ${f}_{sg}$ is the interaction force between the gas phase and the solid phase.

In this paper, the standard turbulence model is used. Since the volume fraction of the sand phase is only 0.02, the turbulent motion of the gas phase plays a dominant role in the wind-blown sand flow. It is assumed that the turbulence develops completely and the influence of the source phase is not taken into account.

(3) Turbulence kinetic energy equation:

5 $$\rho \frac{Dk}{Dt}=\frac{\partial }{{\partial x}_{i}}\left[\left({\mu }_{l}+\frac{{\mu }_{t}}{{\sigma }_{k}}\right)\frac{\delta k}{{\delta x}_{i}}\right]+{G}_{k}+{G}_{b}-\rho \varepsilon .$$

(4) Turbulence dissipation rate equation:

6 $$\rho \frac{D\varepsilon }{Dt}=\frac{\partial }{{\partial x}_{i}}\left[\left(\mu +\frac{{\mu }_{t}}{{\sigma }_{\varepsilon }}\right)\frac{\delta \varepsilon }{{\delta x}_{i}}\right]+{C}_{1\varepsilon }\frac{\varepsilon }{k}\left({G}_{k}+{G}_{3\varepsilon }{G}_{b}\right)-{C}_{2\varepsilon }\rho \frac{{\varepsilon }^{2}}{k},$$

where $\mu ={\mu }_{t}+{\mu }_{l},{\mu }_{t}=\rho {C}_{u}\frac{{k}^{2}}{\varepsilon }$. ${G}_{k}$ is turbulence kinetic energy generated by laminar flow velocity gradient; ${G}_{b}$ is the turbulent kinetic energy generated by buoyancy; $\mu$ is the effective viscosity coefficient; ${u}_{l}$ is laminar viscosity coefficient; ${u}_{t}$ is turbulence viscosity coefficient; ${C}_{1\varepsilon }{,C}_{2\varepsilon },{C}_{3\varepsilon },{\sigma }_{k},{\sigma }_{\varepsilon }$ is the empirical constant, ${C}_{u}$ is the turbulence constant^21^.

#### Boundary definition and computational parameters

The boundary on the left side of the calculation domain was the inlet of wind–sand two-phase flow, which was defined as the boundary condition of the speed inlet. The right side was the outlet boundary of wind–sand flow, which was defined as the boundary condition for the complete development of turbulent outflow. The ground was defined as a nonslip wall and symmetric boundary conditions were adopted at the top of the calculation domain.

The UDF function definition of the wind speed profile at the entrance of the calculation domain was compiled using C language. Combined with the field-measured wind speed data and the *China’s Building Structure Load Code (GB 50,009–2012)*, the empirical formula for the wind speed profile of the Qinghai–Tibet Plateau was used by predecessors, that is, in the vertical direction, the formula is^22^

7 $$V={{V}_{1}\left(Z/{Z}_{1}\right)}^{\alpha },$$

where Z represents any height; the value of Z_1_ is 2 representing 2 m height; V_1_ represents the wind speed at the height of Z_1_; V stands for the wind speed at Z height; α represents the roughness index, according to *Building Structure Load Code (GB 50,009-2012)*, the corresponding value of the ground roughness index α in the Gobi region is 0.2^23^.

#### Fluent for solving the model and setting the parameters

In this study, the unsteady simulation of the wind–sand flow was carried out using the Euler two-fluid model^24^. Wind-blown sand flow is a typical gas–solid flow, and two-phase flow simulation is more reasonable. Euler’s two-fluid model regards particles and fluids as two kinds of fluids, each point in space has its own different speed and density of the two kinds of fluids, these fluids exist in the same space and penetrate each other, but each has different volume fractions and slippage between each other: particle groups interact with gas, and particles interact with each other. The turbulent transport of particles depends on the interphase interaction with gas rather than the interparticle interaction. Each particle phase has a continuous velocity and volume fraction distribution in space. It is more consistent with the actual situation, and the simulation results are more accurate.

In consideration of the eddy current generated when the wind–sand flow passes through the bridge, the turbulence was assumed to be fully developed. The standard k-ε turbulence model was used, and the turbulence intensity (I) is set to 0.02 by calculated. The flow field solving algorithm was adopted by the phase-coupled SIMPLE algorithm which can significantly accelerate convergence. The physical parameters of the model were set as Table 1 ref^11,14,25–28^.

**Table 1:** Specific parameter setting in numerical simulation.

Parameter	Value	Parameter	Value
Underlying surface roughness	0.05	Air density /(kg·m^-3^)	1.225
Subgrade surface roughness	0.08	Air viscosity /(pa·s)	1.6347 × 10^–5^
Turbulence intensity (I)	0.02	Air pressure /pa	56,600
Turbulent dissipation rate	0.5	Sand density /(kg·m^-3^)	2650
Temperature /K	256.95	Average particle size of sand /m	1.25 × 10^–4^
Gravitational acceleration speed /(m·s^-2^)	9.8	Sand phase ratio /%	0.02

## Results of dynamic environment of wind-sand activity

**Figure 6:**
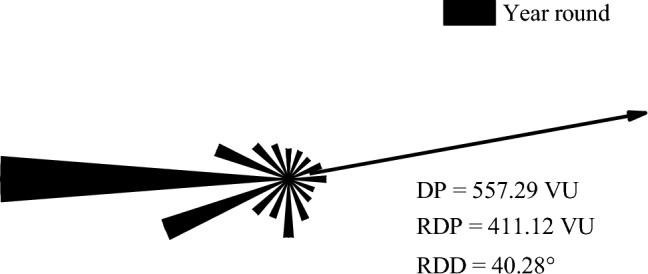
Annual sediment transport potential in the Cuona Lake area.

The average annual wind speed is about 4.12 m/s, and the maximum daily wind speed is 38.0 m/s. The main period of sediment transport is from October to April of the following year, the average wind speed is about 5 m/s in this period. In the meanwhile, the proportion of sand-driving wind from November is the highest. The minimum wind speed is about 3.6 m/s in August and September. The annual resultant sediment transport potential (RDP) of this region is 411.12 VU (≥ 400 VU), which belongs to a high wind energy environment (Fig. 6) .

According to the analysis of the measured wind speed data in the field, the dominant wind direction in the Cuona Lake section is W.The octagonal sediment accumulation instrument (Fig. 2b) showed that the maximum sediment transport in the west direction was 132.2 kg, followed by the E, SE and SW directions, which were 110 kg, 109.4 kg and 97.6 kg respectively. Sand transport in S direction was the smallest, which was 33.64 kg. It further indicates that W is the dominant wind direction。

## Analysis of wind tunnel experiment results

### Influence of wind speed on near-surface flow field on both sides of the bridge

**Figure 7:**
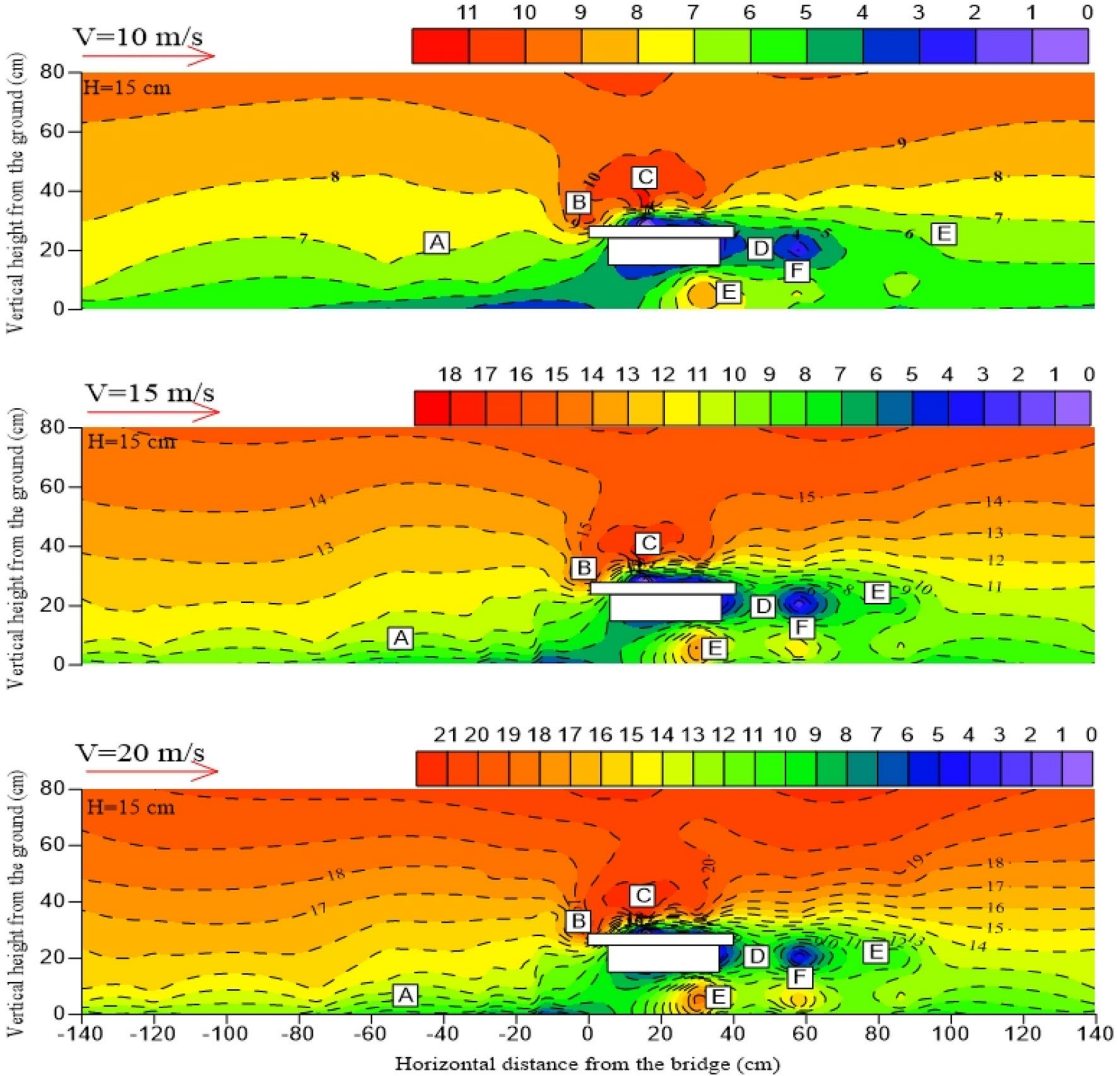
Near-surface flow fields on both sides of the bridge with different incoming wind speeds. (H stands for bridge height in wind tunnel; Negative values on the horizontal axis represent the windward side; Positive values on the horizontal axis represent the leeward side).

**Figure 8:**
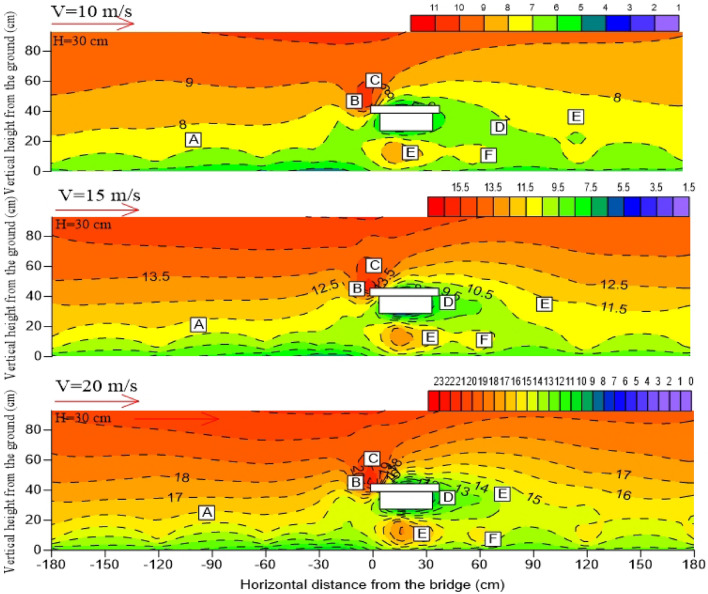
Near-surface flow fields on both sides of the bridge with different incoming wind speeds. (H stands for bridge height in wind tunnel; Negative values on the horizontal axis represent the windward side; Positive values on the horizontal axis represent the leeward side).

When the bridge is at a certain height, the distribution pattern of the surrounding wind speed flow field is the same, including airflow deceleration zone (A), acceleration zone (B), high-speed zone (C), local deceleration zone (D), local acceleration zone (E), and eddy current zone (F)^29–31^. The airflow deceleration zone (A) is distributed on the windward side near the surface, and the wind speed is gradually weakened owing to the influence of the friction resistance of the underlying surface on the windward side. The airflow acceleration zone (B) is mainly distributed in the windward wall surface area of the bridge. The high-speed zone (C) is mainly distributed around 5 – 10 cm above the bridge. The local deceleration zone (D) is mainly distributed in the horizontal direction of the bridge and the leeward side of the bridge. The eddy current zone (F) is mainly distributed on the leeward side of the bridge and part of the bridge. The local acceleration zone (E) exists 2H after the horizontal direction of the leeward side. The wind speed increases at the bottom of the bridge because of the “narrow tube effect” in different degrees, so a local acceleration zone (E) also exists at the bottom of the bridge (Fig. 7 and Fig. 8).

With the increase of incoming wind speed, the values of high-speed zone (C) on the windward side of the bridge increases, and the wind erosion on the windward side of the bridge is strong. In the meanwhile, the wind speed of the bridge bottom clearance area is affected by the “narrow tube effect”, the wind speed increases more significantly, and the sand transport capacity of the bridge bottom is more obvious. However, for the leeward side of the bridge, the expansion of the eddy current zone (F), the increase of the number of vortices, and the longer wind speed recovery distance.

In this paper, the wind speed on both sides of the bridge at the height of 3 cm of the bridge deck is compared and analyzed under the condition of 10 m/s incoming wind speed. The wind tunnel experiment results show that the airflow velocity on the windward side and the leeward side of the 3 m and 6 m high bridges presents a trend of decreasing first and then increasing which is consistent with the variation trend of field-measured wind speed in this area by Xiao Jianhua^32^. The bridge has a great influence on the windward wind speed reduction. On the leeward side, the wind speed attenuates to varying degrees owing to the obstructing effect of the bridge and the diffusion of the air at this section.

**Figure 9:**
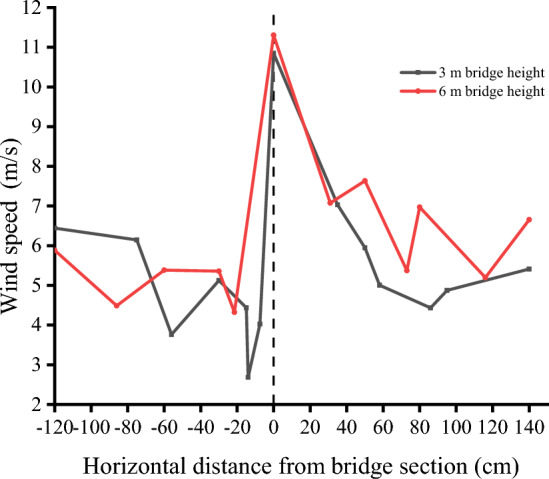
Horizontal distribution of average wind speed.

Bridge height affects the wind speed trough and its location. The horizontal wind speed selected in this paper is the average wind speed measured by pitot tubes at each measuring point on both sides of the bridge at 10 different heights. In the windward side area, the wind speed trough of 3 and 6 m bridges is close to the horizontal distance from the bridge section, but the wind speed trough of the 6 m bridge is higher. Therefore, the windward side of the 6 m bridge has a stronger sand transport capacity and hardly accumulates sand. On the leeward side, the wind speed trough of the 3 m bridge is 80 cm away from the bridge section in the horizontal direction, the wind speed trough of the 6 m bridge is 120 cm away from the bridge, and the wind speed trough of the 6 m bridge is generally higher than that of the 3 m bridge. This difference in flow speed is due to the relatively low weakening degree of airflow speed on the leeward side owing to the increase in bridge height. Coupled with the narrow tube effect the airflow speed near the surface on the leeward side of the 6 m bridge is closer to the incoming wind speed value, which makes the surface sand transport ability stronger. The existence of a wind speed trough is the main reason for sand accumulation on both sides of the bridge. The wind speed troughs on both sides of the bridge with different bridge heights and the horizontal distance from the wind speed trough on the leeward side to the bridge section are compared (Fig. 9). The analysis results show that the sand transport capacity on both sides of the 6 m bridge is stronger.

### Influence of bridge height on near-surface flow field structure

All high-speed areas of bridges are located above bridges, and the higher the height of bridges, the greater the increase of peak wind speed, and the smaller the area of low-speed airflow zone formed on the lee side of bridges. When the wind speed of the 3 m bridge is 20 m/s, the wind speed in the high-speed zone is about 21 m/s, and the wind speed acceleration ratio is 1.05. Under the same incoming wind speed, wind speed in the high-speed zone can reach 23 m/s when the bridge height of 6 m, and the wind speed acceleration ratio is 1.15. It can be seen that the height of the bridge affects the change in wind speed in the high-speed region.

**Figure 10:**
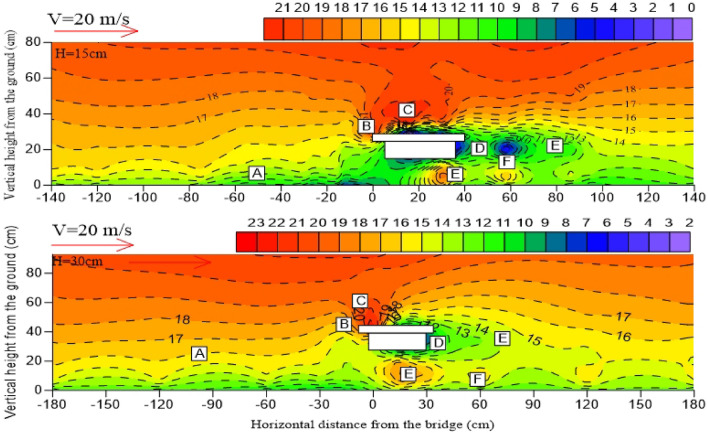
Comparison of flow fields at different bridge heights. (H stands for bridge height in wind tunnel; Negative values on the horizontal axis represent the windward side; Positive values on the horizontal axis represent the leeward side).

The bridge height determines the distribution location and range of eddy current on the leeward side. The eddy current on the bridge with a height of 3 m is mainly distributed at the upper interface of the leeward side, whereas the eddy current on the bridge with a height of 6 m is mainly distributed on the leeward side near the surface. The area of the bridge vortex zone also changes with the bridge height. The higher the bridge is, the smaller the area of the vortex zone will be. When the airflow in the high-speed zone moves to the leeward side, the upper airflow speed in this area is slow because the 3 m bridge height has a large deceleration amplitude on the airflow in the deceleration zone, and the overhead area at the bottom of the bridge flows into the leeward side near the ground owing to the “narrow tube effect” (Fig. 10). According to Bernoulli's principle, the pressure difference between the upper and lower interfaces on the leeward side is large. That is, the eddy current zone on the leeward side of the 3 m high bridge is more obvious, and the range is larger. The bridge height of 6 m has a small weakening range on the upper airflow speed on the leeward side, and the pressure difference between the upper and lower layers on the leeward side is not large. Hence, the eddy current zone on the leeward side of the 6 m high bridge is not obvious.

### Characteristics of near-surface wind speed profiles around bridges

**Figure 11:**
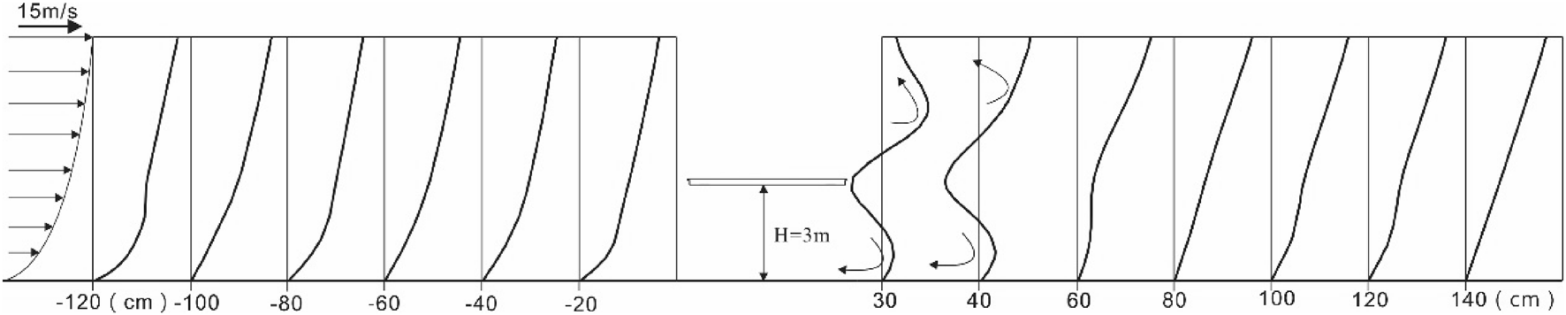
Wind speed profile of each measuring point on the bridge cross-section.

The wind speed profile is the curve of wind speed distribution along with height, which is an important way to show the change in airflow structure. The wind speed profile on the windward side conforms to the wall law. However, owing to the existence of friction resistance on the underlying surface, when the airflow gradually approaches the bridge section, the bottom of the wind speed profile gradually deviates from the logarithmic distribution, and the wind speed near the ground shows a decreasing trend (Fig. 11). On the leeward side of the bridge, because of the influence of the bridge section, the airflow no longer follows the wall law, and the wind speed profile changes significantly, showing considerable linear characteristics. It gradually recovers to the initial state after 100 cm downwind.

**Figure 12:**
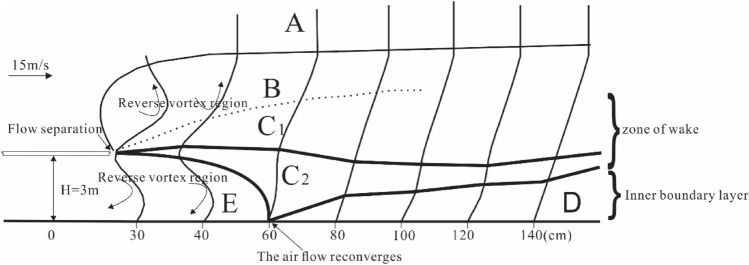
Secondary flow zoning on the leeward.

When studying the airflow on the leeward side of the dune, predecessors found that the airflow pattern on this side was the result of the interaction between the dune and the original airflow. Therefore, the airflow pattern changed by the action of the dune was called secondary flow. Its airflow movement and energy distribution were quite different from those of the original airflow^33^. According to the non-log-linear wind speed profile obtained from the wind tunnel experiment, the different wind speed regions are divided by combining with the speed gradient. The airflow on the leeward side of the bridge can also be divided into five zones: outflow zone (A), overflow zone (B), wake zone (C), boundary layer (D), and reverse vortex zone (E). The five zones show a gradual transition with different ranges (Fig. 12).

In the outflow zone (A), the wind speed gradient varies slightly with the height, and the airflow movement in this zone is least affected by the bridge section. From the wind speed profile of the leeward side, an obvious speed inflection point exists between this zone and the overflow zone of the lower layer. Within the overflow zone (B) range, the speed gradient in the wind speed profile is characterized by a large variation, which is formed by the “ejection” effect of the high-speed airflow. With the development of the downwind direction, the speed gradient also decreases correspondingly and finally merges into the wake zone. The wake zone mainly consists of C_1_ and C_2_. The wind speed and speed gradient in the C_1_ zone are both larger than those in the C_2_ zone, and the wake zone eventually merges with the downwind development of the two zones. The inner boundary layer (D) region represents the magnitude of shear force and its range of influence. This region starts from the reconvergence point of airflow. The more downwind, the thicker the boundary layer is, and the stronger the acceleration effect of shear force on airflow is. The appearance of reverse eddy current region E is mainly influenced by the vertical and high-speed incident airflow on the windward side. In accordance with the principle of low air pressure where the speed is high, a low-pressure region appears on the leeward side, which leads to the reverse movement of airflow. The speed gradient in this region is small, and it is close to the bridge^2^. The shear effect of airflow on the surface is correspondingly small.

## Analysis of numerical simulation results

### Flow field verification

**Figure 13:**
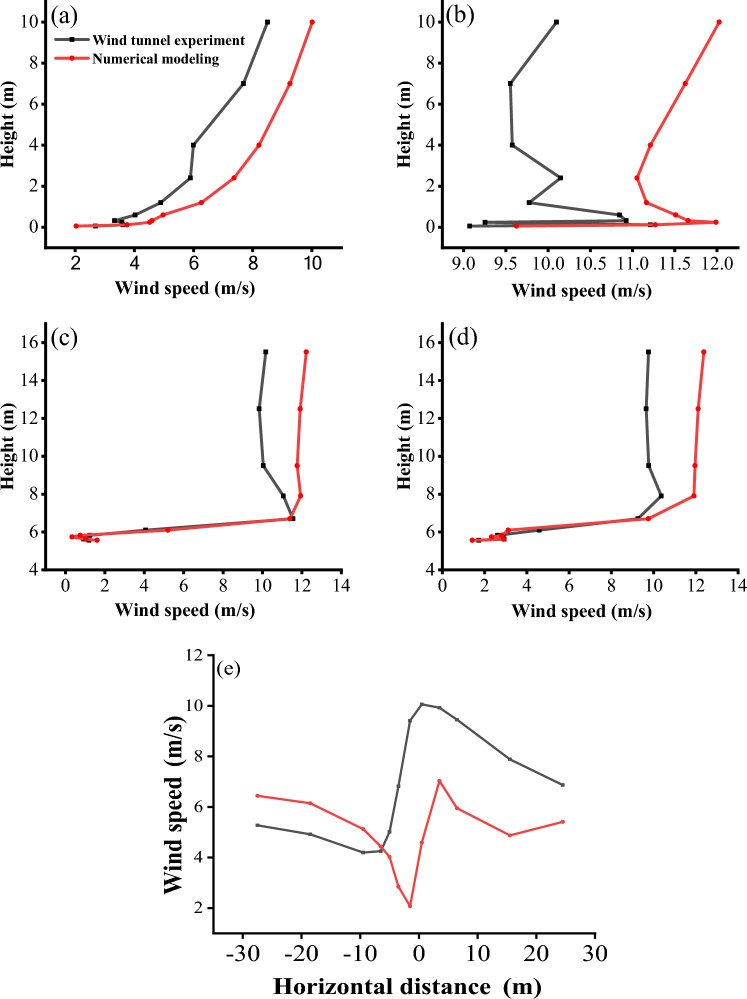
Comparison of wind tunnel experiment and numerical simulation wind profile. (**a**): Entrance; (**b**): Windward side of the bridge; (**c**): Middle of the bridge; (**d**): Leeward side of the bridge; (**e**): Wind speed in the horizontal direction).

To ensure the reliability of the numerical simulation results, the same initial conditions were set in the numerical simulation as in the wind tunnel experiment. Taking the wind speed of incoming flow of 10 m/s as an example, comparing the wind speed profile at the entrance, windward side of the bridge, the center of the bridge, and the wind speed at the same height in the horizontal direction, it can be seen that the wind speed results of the two are much the same (Fig. 13). It shows that the flow field is set reasonably in the numerical simulation, and then the numerical simulation flow field is applied to the calculation of the flow field around the bridge with different heights.

### Analysis of sand damage formation mechanism

Aeolian sand flow is a kind of gas–solid two-phase flow. It is the wind carrying sand grains to move, and the motion state of sand grains is closely related to the wind speed. The speed changes on both sides of the bridge and in the overhead area at the bottom of the bridge can reflect the motion state of the wind-blown sand flow and the forms of sand damage^34–36^. The height of the bridge affects the distribution characteristics of the flow field, which further influences the change in regional speed and leads to the change in the sand particle motion state. As the wind speed in some areas of the flow field is reduced, the sand-carrying capacity of the airflow is weakened. This phenomenon directly leads to the large-scale accumulation of sand particles, which is the most important reason for the formation of sand burying damage. According to the analysis of the distribution characteristics of the bridge flow field at different heights simulated by wind tunnel experiments, the phenomenon of bridge sand deposition mainly appears on the windward side, the leeward side, the low-wind-speed area, the eddy current area, and the bridge bottom overhead area. In particular, the bridge bottom area is influenced by the “narrow tube effect” but it still exists under certain wind conditions.

In consideration of the sand-driving wind speed, the wind speed of the initial flow was set to 5 m/s for example. While the volume fraction of sand particle is to 0.63, it is the state of sand accumulation in the color map. In this paper, the sand accumulation at the bottom of the bridge is illustrated by local magnification.

**Figure 14:**
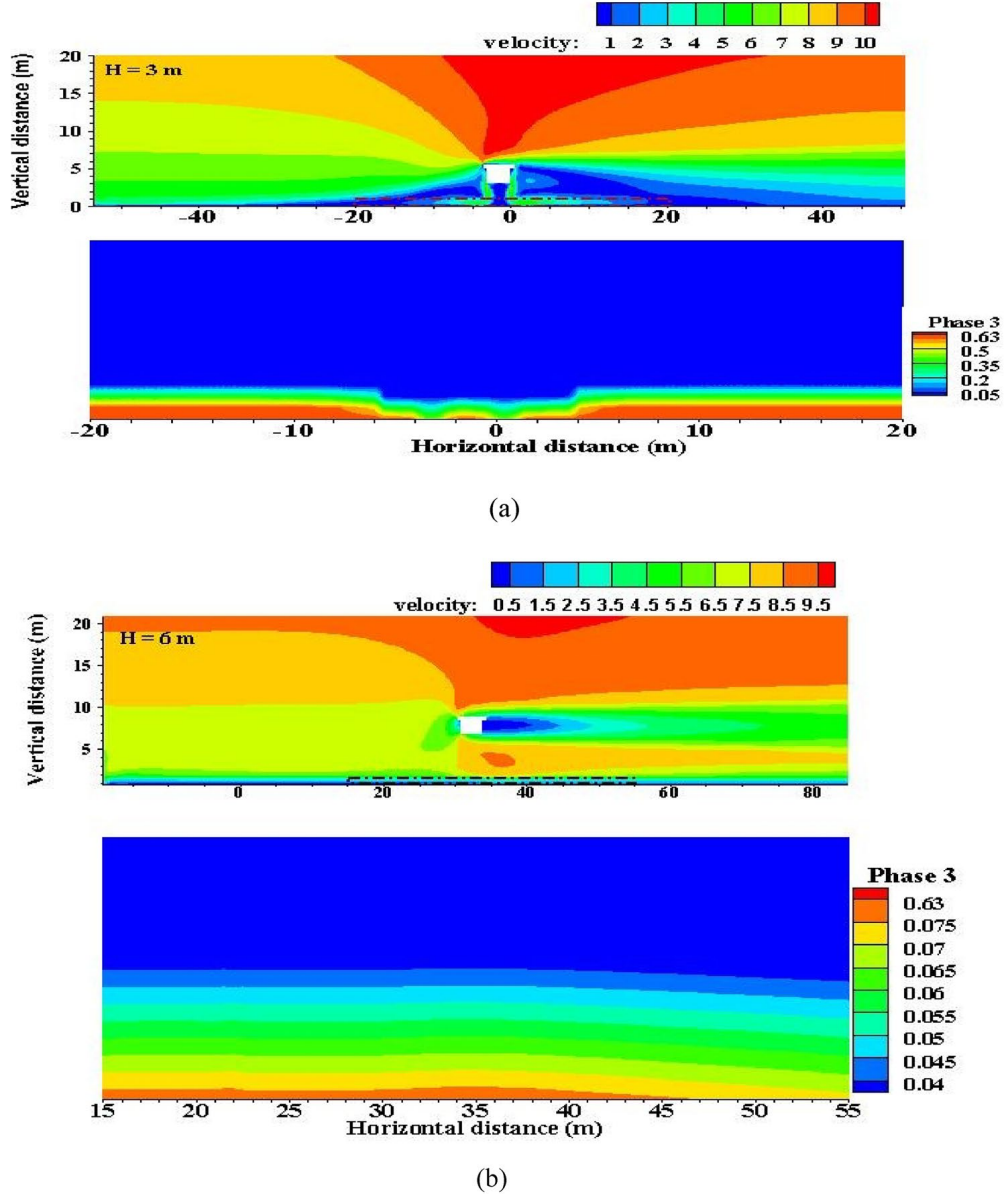
Distribution of wind speed and sand accumulation at different bridge heights. (H stands for bridge height in numerical simulation).

The sand accumulation on the windward side of the bridge is due to the rough underlying surface, which consumes the energy of the wind-blown sand flow and leads to a decrease in wind speed (Fig. 14a). The sand particles in the near-surface movement of “creep” and “saltation” gather on the windward side of the bridge in the way of “drop deposition”. The phenomenon of sand deposition on the leeward side of the bridge is caused by the sharp reduction in wind speed caused by the airflow in the reverse eddy current zone on the leeward side, as well as the large amount of sand accumulation in the supersaturated aeolian sand flow owing to the significant reduction in the airflow's sand-carrying capacity. For the overhead area at the bottom of the bridge, because the wind speed in this area is lower than the sand-driving wind speed under certain wind conditions, the sand particles trapped in the previous wind-driven sand flow gradually accumulate on the surface (the volum fraction of most sand particles is close to 0.63). In addition, the airflow line in this area is not parallel to the surface distribution; some sand particles collide with the beam body under the action of airflow and then fall on the ground, resulting in sand accumulation^37–39^.

When the bridge reaches a certain height, the flow field structure and speed at the bottom of the bridge change (Fig. 14b). Under the same incoming wind speed, the gap between the wind speed at the bottom of the bridge and the wind speed of the incoming flow decreases, the sand transport capacity at the bottom of the bridge is enhanced (the volum fraction of sand partical is mostly lower than 0.63), the wind-blown sand flow can normally pass through the bottom of the bridge and no longer generates accumulation, and the influence of the bridge height on the balance state of wind-blown sand flow is eliminated. Comparison of wind speed trough values on both sides of the bridge shows that the increase in bridge height enhances the sand transport capacity on both sides^40–42^.

## Conclusions

1. The results of dynamic environment of wind-sand activity show that the west wind is the main sand-driving wind in Cuona Lake area. The average wind speed from November to April of the following year is about 5 m/s, and the minimum wind speed is about 3.6 m/s in August and September. The annual resultant sediment transport potential (RDP) of this region is 411.12 VU.
2. Near-surface flow fields on both sides of the bridge is divided into the windward airflow deceleration zone, acceleration zone, high-speed zone, eddy current zone, local deceleration zone, and local acceleration zone. Under the influence of the narrow tube effect, a local acceleration zone also exists at the bottom of the bridge.
3. The secondary flow zone on the leeward side of the bridge can be divided into five areas: reverse vortex zone, outflow zone, overflow zone, wake zone (including upper and lower wake zones), and inner boundary layer. The reverse vortex zone easily causes sand accumulation on the leeward side of the bridge.
4. Under certain wind speed conditions, the overhead area at the bottom of the 3 m high bridge and its two sides do not have the sand transport capacity and are prone to sand accumulation. Nevertheless, the sand accumulation phenomenon gradually disappears with the increase in bridge clearance height, so the bridge height in the leeward area should not be less than 6 m.
5. The west side of Basuoqu bridge needs to be laid key engineering sand prevention measures. It is advisable to lay several vertical sand barriers in the area beyond 10 m on the west side of the bridge, whose direction is parallel to the direction of the bridge; On the side near the bridge, gravel grid can be laid or gravel compaction can be used to effectively reduce the sand-carrying capacity of the sand-carrying wind. At the same time, the area near Cuona Lake on the west side of the bridge can be blocked by water storage and sand compaction to prevent local sand generation. For the east side of the bridge, it is not appropriate to set up more construction machinery protection measures in this area, it is necessary to focus on the restoration of vegetation in the area, which is more suitable for plant sand fixation.

## Data Availability

All data generated or analysed during this study are included in this published article.
